# Tocilizumab combined with short-term high-dose glucocorticoids for rapid disease activity control and glucocorticoid reduction in adult-onset Still’s disease: a single-center retrospective study

**DOI:** 10.3389/fimmu.2026.1732826

**Published:** 2026-02-05

**Authors:** Shi-Lei Zhong, Yan-Ping Lei, Li-Xuan Zhou, Ai-Xia Niu, Bo Liu, Yong-Long He, Qi-Bin Yang

**Affiliations:** 1 Department of Rheumatology and Immunology, Affiliated Hospital of North Sichuan Medical College, Nanchong, Sichuan, China; 2 Graduate School of North Sichuan Medical College, Nanchong, Sichuan, China

**Keywords:** adult-onset still’s disease, biologic therapy, glucocorticoids, retrospective studies, tocilizumab, treatment outcome

## Abstract

**Objectives:**

This study aimed to evaluate the effectiveness of tocilizumab (TCZ) combined with short-term high-dose glucocorticoids in rapidly controlling disease activity and reducing glucocorticoid use in patients with adult-onset Still’s disease (AOSD).

**Methods:**

In this single-center retrospective study, all patients meeting the inclusion criteria were enrolled. Patients receiving short-term high-dose glucocorticoids (5 mg/kg/day for 3 days) with TCZ (400 mg every 4 weeks) were assigned to the TCZ group, while those receiving standard-dose glucocorticoids (1 mg/kg/day) without TCZ formed the non-TCZ group. Efficacy was evaluated based on laboratory data, clinical and Pouchot scores. Glucocorticoids -retention rate was estimated by the Kaplan-Meier method.

**Results:**

Fifty patients (11 men, 39 women) were included (19 in the TCZ group and 31 in the non-TCZ group). The TCZ group had a higher incidence of skin rash (100% vs 58.1%, *P* = 0.003) and sore throat (84.2% vs 29.0%, *P* < 0.001). Over the follow-up period, inflammatory markers (CRP, ESR, SF), liver enzymes (AST, ALT), and WBC counts significantly declined in the TCZ group (*P* < 0.05). Remission rates were higher in the TCZ group at months 1 (63.2% vs 9.7%), 3 (88.9% vs 20.0%), and 6 (83.3% vs 25.9%) (*P* < 0.001). Despite a higher initial glucocorticoid dose, no significant differences in subsequent doses were observed between groups. By 36 months, the TCZ group showed a significantly higher glucocorticoid discontinuation rate (77.0% vs 30.9%, *P* = 0.0046). TCZ treatment was also associated with improved liver function indicators and reduced liver injury (10.5% vs 32.3%).

**Conclusion:**

Tocilizumab combined with short-term high-dose glucocorticoids may provide rapid disease control and facilitate glucocorticoid tapering in AOSD. More prospective studies are needed to confirm these findings.

## Introduction

Adult-onset Still’s Disease (AOSD) is a rare systemic inflammatory disorder with an unclear etiology and a wide spectrum of clinical manifestations. Primarily affecting individuals aged 16–35 years, its global incidence ranges from 0.16 to 0.4 cases per 100,000 individuals annually, depending on population and region (1–8). Diagnosis is often delayed by 1.5–4 years from symptom onset, relying on the classic triad of abrupt high fever, arthritis/arthralgia, and evanescent rash, alongside hyperferritinemia. Additional features may include leukocytosis, elevated liver enzymes, hepatosplenomegaly, lymphadenopathy, elevated inflammatory markers, and potentially life-threatening complications, with macrophage activation syndrome (MAS) emerging as the most severe. The prevalence of MAS ranges between 10 and 15%, and it is associated with a high mortality rate (1).

The pathogenesis of AOSD remains incompletely understood but involves genetic predisposition (e.g., HLA-Bw35, HLA-B17, HLA-B18, HLA-B35, HLA-DR4, HLA-DR2, HLA-DRw6 and HLA-DRB1), infectious triggers (e.g., Parvovirus B19, Epstein-Barr virus, Cytomegalovirus), and dysregulated innate immunity, particularly macrophage and neutrophil activation. Macrophages drive systemic inflammation via excessive production of pro-inflammatory cytokines, including interleukin (IL)-6, IL-1β, and TNF-α, which amplify neutrophil recruitment and endothelial activation. Among these, IL-6 is a central mediator: it upregulates acute-phase reactants (e.g., ferritin, CRP), promotes B-cell differentiation, contributing to both systemic inflammation and end-organ damage. Notably, IL-6 levels are closely associated with clinical manifestations of AOSD (e.g., salmon-colored skin rash, arthralgia) and disease activity (1–3, 9–13).

Clinically, patients exhibit either predominant systemic symptoms or prominent arthritis. The disease course is classified into three patterns: monocyclic (single self-limiting episode), polycyclic (recurrent symptomatic flares), or chronic (persistent symptoms beyond 1 year) (1, 3, 5, 8, 10, 14). Management remains challenging due to disease heterogeneity and the risk of refractory inflammation or treatment-related toxicity. The current first-line therapy involves systemic glucocorticoids (0.5–1.0 mg/kg/day), often combined with conventional synthetic disease-modifying antirheumatic drugs (csDMARDs) to control disease activity (1, 15). However, long-term GC use exposes patients to cumulative GC-related adverse effects, including osteoporosis, diabetes mellitus, and opportunistic infections. The csDMARDs have been shown to induce remission in approximately 80% of AOSD patients (1). Methotrexate (MTX), for instance, has demonstrated efficacy in reducing glucocorticoids dosage while inducing remission (9, 16). However, csDMARDs are limited by organ toxicity (e.g., MTX-induced hepatotoxicity, leflunomide-related teratogenicity) and variable efficacy, necessitating alternative strategies (1, 3). More studies are needed to elucidate AOSD pathophysiology and to refine personalized therapeutic approaches.

In recent years, the application of biologics such as IL-1 inhibitors (e.g. anakinra) and IL-6 inhibitors (e.g. tocilizumab, TCZ) has revolutionized the management of AOSD. These inhibitors have demonstrated efficacy in inducing remission and reducing glucocorticoid (GC) requirements (5, 9, 12, 14, 17–19). TCZ, a humanized anti-IL-6 receptor antibody, blocks IL-6 signaling to inhibit downstream inflammatory pathways, demonstrating marked efficacy in alleviating clinical symptoms and reducing steroid dependency (1, 7, 14, 16, 20, 21).

Multiple studies have demonstrated that TCZ alleviates systemic and articular symptoms, reduces GC dosage, and yields promising outcomes in refractory AOSD (1, 3, 14, 19–22). A randomized, double-blind, placebo-controlled trial further showed that a higher proportion of patients treated with TCZ achieved an ACR50 response by week 4 (20). Despite these advancements, critical gaps persist. Firstly, optimal GC dosing strategies—specifically, the choice between short-term high-dose GCs (e.g. 5 mg/kg/day for 3 days) and standard daily dosing—remain controversial. While short-term high-dose GCs can rapidly suppress inflammation, robust clinical evidence supporting their use in AOSD, particularly when combined with biologics like TCZ, is lacking. Secondly, no study has yet evaluated the efficacy of short-term high-dose GCs combined with TCZ (400 mg every 4 weeks) versus standard GCs regimens (1 mg/kg/day) without TCZ. This gap limits our capacity to optimize treatment strategies and mitigate long-term GCs exposure. Short-term high-dose GCs rapidly suppress multiple inflammatory pathways, creating a therapeutic window for TCZ to halt disease progression. This approach may promote sustained remission while reducing cumulative GCs exposure. Therefore, this retrospective study aimed to evaluate the efficacy and safety of a combined induction strategy of TCZ with short-term high-dose GCs in AOSD patients and to compare it with a conventional strategy of csDMARDs combined with standard-dose GCs. We hypothesized that the combination of TCZ and short-term high-dose GCs would control disease activity more rapidly, reduce cumulative GC exposure and improve clinical outcomes compared to standard therapy.

## Materials and methods

### Enroll criteria of AOSD patients and medication treatment

**Figure 1:**
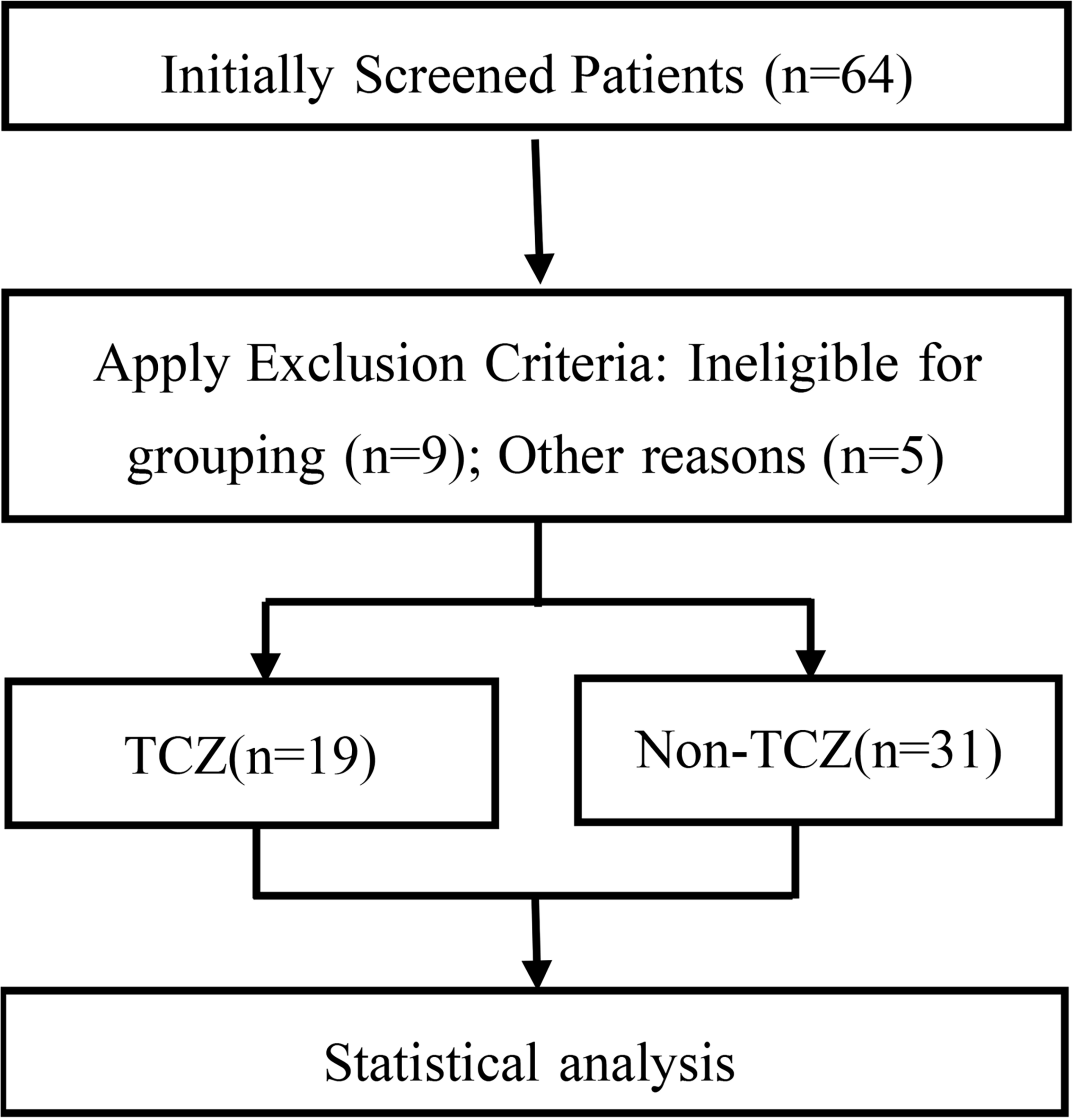
Flowchart of patients selection.

All patients diagnosed with AOSD at the Affiliated Hospital of North Sichuan Medical College from January 2014 to May 2024 were enrolled. Patients included had to be 16 years old or older, to be diagnosed with AOSD, to meet the Yamaguchi criteria (23). Primary exclusion criteria encompassed individuals under 16 years, those with systemic juvenile idiopathic arthritis (SoJIA), failure to meet Yamaguchi diagnostic criteria, an exclusion of AOSD diagnosis during disease course, and exclusion of cases missing >20% of key indicators (17) and cases with incompatible treatment regimens [i.e., receiving TCZ but not short-term high-dose GCs (n=4), receiving short-term high-dose GCs but not TCZ (n=2), and cases using other biologics (such as IL-1 inhibitors, n=3)] as shown in **Figure 1**. Given the unavailability of glycosylated ferritin assays, the Yamaguchi criteria were adopted despite the Fautrel criteria offering greater specificity (5). Given the small-sample retrospective nature of the study, to avoid overprocessing, simple imputation methods were applied for those with missing values between 5% and 20%. In this study, 19 patients received short-term, high-dose intravenous glucocorticoids (5 mg/kg/day for 3 days, followed by maintenance prednisone ≤ 1 mg/kg/day) alongside tocilizumab (400 mg every 4 weeks), constituting the TCZ group. Conversely, 31 patients were administered standard glucocorticoids therapy (1 mg/kg/day) combined with csDMARDs therapy without tocilizumab, forming the non-TCZ cohort. Disease status was categorised as newly diagnosed or relapsed cases based on the initial condition at the start of treatment. Relapse was defined as a previous diagnosis of AOSD followed by systemic treatment and subsequent disease reactivation. As this was a retrospective study, patients were not randomly assigned to the TCZ or non-TCZ group but allocated based on multidisciplinary clinical judgement. TCZ combined with short-term high-dose GCs was exclusively reserved for AOSD patients with poor response to conventional therapy—defined as uncontrolled disease activity, persistent elevations in inflammatory markers (e.g., CRP, ESR), or recurrent disease flares. Patients performed a rheumatologic evaluation (Pouchot score). Clinical and laboratory parameters were retrospectively collected at baseline and months 1, 3, 6, 9, and 12. To investigate the long-term effects of glucocorticoid tapering and discontinuation, glucocorticoid use was followed up for 36 months. The primary efficacy endpoint was remission, which was defined as the achievement of clinical inactive disease (CID), which required the concurrent fulfillment of all the following criteria: complete absence of AOSD-related clinical symptoms (e.g., fever, rash, arthralgia), either ESR ≤ 26 mm/h or CRP ≤ 10 mg/L, a serum ferritin ≤ 5 times the upper limit of normal (233 ng/mL), and AST and ALT levels ≤ 1.5 times the upper limit of normal (35U/L, 40U/L, respectively). Relapse was declared when both of the following conditions were met: reemergence of clinical symptom (e.g., high fever, typical rash, active arthritis) and at least one key laboratory marker (ESR ≥26 mm/h, CRP ≥10 mg/L, or serum ferritin ≥ 5 times the upper limit of normal). This research study was approved by the Affiliated Hospital of North Sichuan Medical College Ethics Board (IRB: 2024ER456-1). All individuals have provided written informed consent for this research study.

### Clinical and laboratory data collection

Data collection included patient demographics (gender, age), clinical manifestations (fever, rash, arthralgia/arthritis, lymphadenopathy, sore throat, splenomegaly, hepatomegaly, infection, hypertension, diabetes, gastrointestinal symptoms, etc.), and laboratory findings [blood/bone marrow cultures, PET/CT examinations, bone marrow biopsy, lymph node biopsy, antinuclear antibodies (ANA), anti-citrullinated peptide autoantibodies (anti-CCP), rheumatoid factor (RF), leukocytes (WBC), neutrophil percentage (N%), erythrocyte sedimentation rate (ESR), C-reactive protein (CRP), serum ferritin (SF), aspartate aminotransferase (AST) and alanine aminotransferase (ALT)]. For Pouchot score of disease activity (24), it was recorded only if symptoms occurred within the specified assessment period. The Pouchot score at baseline assessment records all relevant symptoms that occurred from the onset of the current disease episode to the baseline assessment, reflecting the cumulative burden of the current episode. The Pouchot score at the 1-month follow-up recorded new or persistent symptoms that occurred from the baseline assessment to the 1-month follow-up, thus assessing disease activity during that period. Treatment details (glucocorticoids, csDMARDs, and tocilizumab dosages), disease activity, adverse events and the cumulative MTX dose were also recorded.

### Statistical analysis

The variables were tested for normality using the Shapiro-Wilk test. Continuous variables were expressed as mean ± standard deviation if normally distributed, or as median (interquartile range) otherwise. The categorical variables were expressed as percentages. Between-group differences for quantitative variables at each follow-up time point were assessed using independent samples t-tests for normally distributed data and Mann–Whitney U tests for non-normally distributed data. Categorical variables were compared using chi-squared or Fisher’s exact tests, as appropriate. Longitudinal trends in laboratory parameters (e.g., CRP, ESR) across follow-up time points were evaluated via repeated-measures ANOVA. Glucocorticoids -retention rate was estimated by the Kaplan-Meier method. SPSS 27.0 and GraphPad Prism 9.5 were used for statistical analysis, and P<0.05 was considered statistically significant.

The analysis focuses on patients who had data available at the time of treatment. Laboratory parameters in the TCZ group were analyzed by comparing data from the months of follow-up. Differences in laboratory parameters between the two groups at different follow-up time points were also compared, as well as the rates of disease remission and adverse events in the two groups.

## Results

### Characteristics of patients with AOSD

A total of 50 patients (11 men, 39 women) were enrolled in this study from January 1, 2014, to May 31, 2024; however, 14 patients were excluded. Demographic, clinical, and laboratory data for all participants are summarized in **Table 1**. The median age was 29 years [IQR: 24.00, 49.00] in the TCZ group, compared to 43 years [IQR: 32.00, 48.00] in the non-TCZ group. Both cohorts showed a female predominance, with 68.4% of TCZ patients and 83.9% of non-TCZ patients being female (*P*>0.05). Clinical features revealed no significant differences except for skin rash and sore throat. In the TCZ group, 100% of patients exhibited skin rash (n=19) and 84.2% reported sore throat (n=16), significantly higher than in the non-TCZ group. There was no significantly difference in disease status (new diagnosis and relapse) between the two groups at baseline, making them comparable in terms of the potential bias factor of disease refractoryness. Laboratory findings indicated elevated levels of ESR, WBC, CRP, SF, AST, and ALT, exceeding normal ranges across both cohorts. Overall, fever, arthralgia, and skin rash were prevalent symptoms in both the TCZ and non-TCZ groups. In the TCZ group, the first TCZ infusion was initiated either concomitantly with or within 72 hours of high-dose glucocorticoid therapy onset, with a median interval of 1 day [IQR: 1, 1]. All patients thus received early combination immunomodulatory intervention. During the 12-month follow-up, 15 patients (78.95%) maintained continuous TCZ therapy, reflecting favorable treatment persistence and disease control. Discontinuation occurred in 4 patients: 3 achieved sustained remission for at least six months off therapy, while 1 discontinued due to financial limitations.

**Table 1:** Baseline characteristics of patients with adult-onset Still’s disease.

Characteristics	Treatment with tocilizumab group (n=19)	Treatment without tocilizumab group (n=31)	*P* value
Age (years*)	29[24.00,49.00]	43.00[32.00,48.00]	0.144
Gender, female, n (%)	13(68.4)	26(83.9)	0.293
Fever, n (%)	19(100)	30(96.8)	1.000
Arthralgia, n (%)	13(38.4)	22(71.0)	0.849
Skin rash, n (%)	19(100)	18(58.1)	0.003^#^
Sore throat, n (%)	16(84.2)	9(29.0)	<0.001^#^
Lymph node enlargement or hepatomegaly or splenomegaly, n (%)	7(36.8)	5(16.1)	0.171
Relapsed disease, n (%)	8(42.1)	16(51.6)	0.514
Newly diagnosed, n (%)	11(57.9)	15(48.4)	0.514
ESR (mm/h)	107.74±134.53	59.90±28.24	0.069
WBC (×10^9^/L)	22.83±9.24	17.73±6.38	0.055
CRP (mg/L)	125.95±73.07	86.39±66.42	0.075
SF (μg/L)	1433.78±524.06	1090.81±809.14	0.096
AST (U/L)	59.33±55.39	61.26±79.47	0.929
ALT (U/L)	72.93±83.72	54.77±99.97	0.532
ANA, n(%)	3(15.8)	6(19.4)	1.000
RF, n(%)	2(10.5)	3(9.7)	1.000
Neu (%)	82.19±6.94	78.44±11.48	0.233
Neu (×10^9^/L*)	12.69[11.36,16.30]	12.52[7.38,14.43]	0.172

*Median with the p25–p75 between brackets.

^#^Statistically significant result (*P*<0.05).

ESR, erythrocyte sedimentation rate; WBC, leukocyte; CRP, C-reactive protein; SF, serum ferritin; AST, aspartate transaminase; ALT, alanine aminotransferase; ANA, antinuclear antibody; RF, rheumatoid factor; Neu%, neutrophil percentage; Neu, neutrophil.

### Short-term high doses of glucocorticoids combined with TCZ rapidly reduce inflammatory markers in AOSD

**Figure 2:**
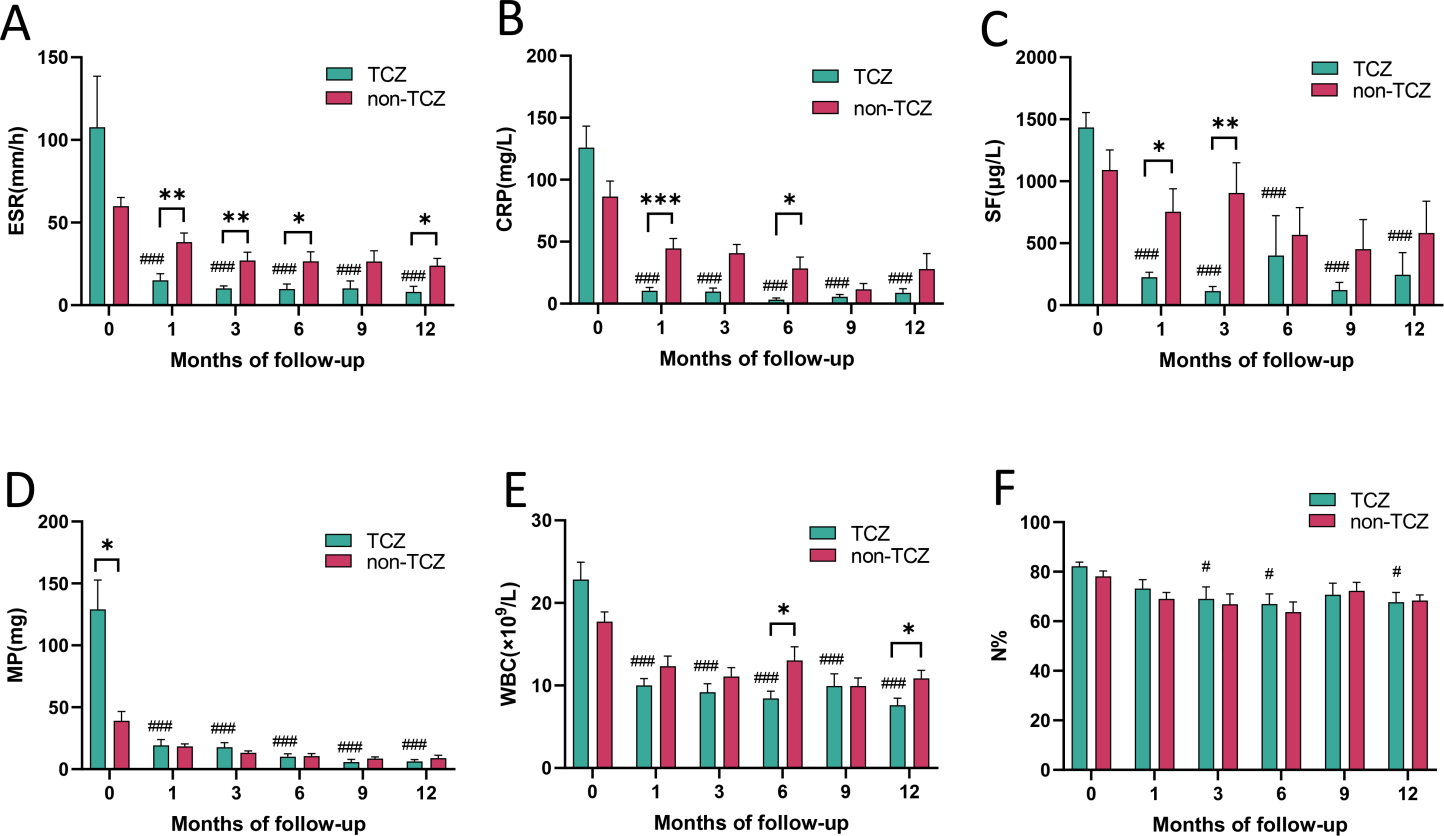
Comparison of laboratory indicators and glucocorticoid dosage before and after treatment in the TCZ group and between the TCZ group and the non-TCZ group. ^*^P < 0.05, ^**^P < 0.01, ^***^P < 0.001; ^#^*P* < 0.05, ^##^*P* < 0.01, ^###^*P* < 0.001.

Laboratory parameters in AOSD patients treated with short-term high doses of GCs (methylprednisone, MP) combined with TCZ are depicted in **Figure 2**. In the TCZ group, significant reductions in CRP (**Figure 2B**), SF (**Figure 2C**), and WBC (**Figure 2E**) were observed at various follow-up intervals compared to baseline (*P* < 0.001, respectively). These markers demonstrated marked decreases by the first month (*P* < 0.001) and remained suppressed over time. Similarly, in addition to MP (**Figure 2D**), ESR (**Figure 2A**) levels exhibited significant declines across all follow-up points (*P* < 0.001), while percentages of neutrophils (**Figure 2F**) marginally decreased without statistical significance during the initial month. Comparative data between TCZ and non-TCZ groups were aslo illustrated in **Figure 2**. At one-month follow-up, ESR (**Figure 2A**, *P* < 0.01), CRP (**Figure 2B**, *P* < 0.001), and SF (**Figure 2C**, *P* < 0.05) levels were notably lower in the TCZ group. By six months, TCZ-treated patients maintained significantly reduced ESR (**Figure 2A**), CRP (**Figure 2B**) and WBC (**Figure 2E**) values relative to non-TCZ groups (*P* < 0.05, respectively). However, MP (**Figure 2D**) and the percentages of neutrophils (**Figure 2F**) always kept the similar between two groups at various follow-up intervals.

### Short-term high doses of glucocorticoids combined with TCZ rapidly induce clinical remission in AOSD

**Figure 3:**
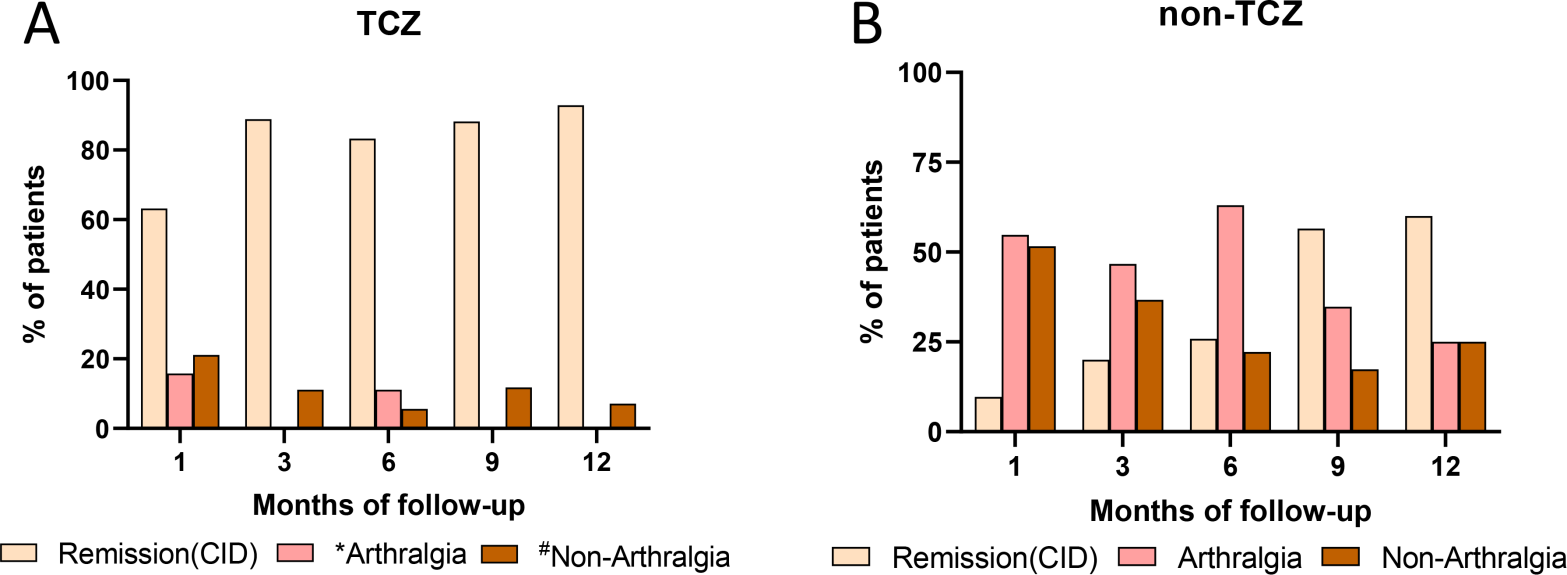
Remission (CID) and clinical manifestations were showed at each follow-up time points in the TCZ group and non-TCZ group. *Percentage of patients with unresolved symptoms exhibiting arthralgia. ^#^Percentage of patients with unresolved symptoms exhibiting non-arthralgia.

**Table 2** summarized the Pouchot scores for patients in both the TCZ and non-TCZ groups from baseline assessment to one-month follow-up. A significant reduction in Pouchot scores was observed at one-month post-baseline assessment (*P* < 0.05). The TCZ group (**Figure 3A**) exhibited substantially higher remission rates compared to the non-TCZ group (**Figure 3B**) at one (63.2% vs 9.7%, *P* < 0.001), three (88.9% vs 20.0%, *P* < 0.001), six (83.3% vs 25.9%, *P* < 0.001), and nine months (88.2% vs 56.5%, *P* < 0.05) of follow-up, as depicted in **Figure 3**. However, no significant difference in remission rate was noted between the two groups at twelve months (*P*>0.05). At the one-month follow-up, the TCZ group achieved a remission rate exceeding 50%, whereas the non-TCZ group did not reach 50% remission rate until the ninth month. Moreover, patients who did not achieve remission in the tocilizumab group typically presented with non-articular pain symptoms or a few arthralgia symptoms (**Figure 3**).

**Table 2:** Clinical and laboratory items at baseline and the month 1 of follow-up assessment for the Pouchot score in the TCZ group and the non-TCZ group.

Items	Treatment with tocilizumab group (n=19)		Treatment without tocilizumab group(n=31)	
	Baseline (n. of patients)	Month 1 of follow-up (n. of patients)	Baseline (n. of patients)	Month 1 of follow-up (n. of patients)
Fever	19	1	30	12
Skin rash	19	4	18	5
Sore throat	16	0	9	1
Lymphadenopathy	8	0	4	1
Myalgia	14	3	24	17
Pleuritis	0	0	0	0
Pericarditis	0	0	0	0
Leukocytosis>15 000/mm^3^	15	2	23	4
Hepatomegaly or abnormal liver function	9	2	19	9
Splenomegaly	0	0	1	0
Abdominal Pain	0	0	0	0
Change of Pouchot***	4.00(1.00)		3.00(1.00)	

***P<0.001.

### Comparison of disease recurrence and glucocorticoid discontinuation

**Figure 4:**
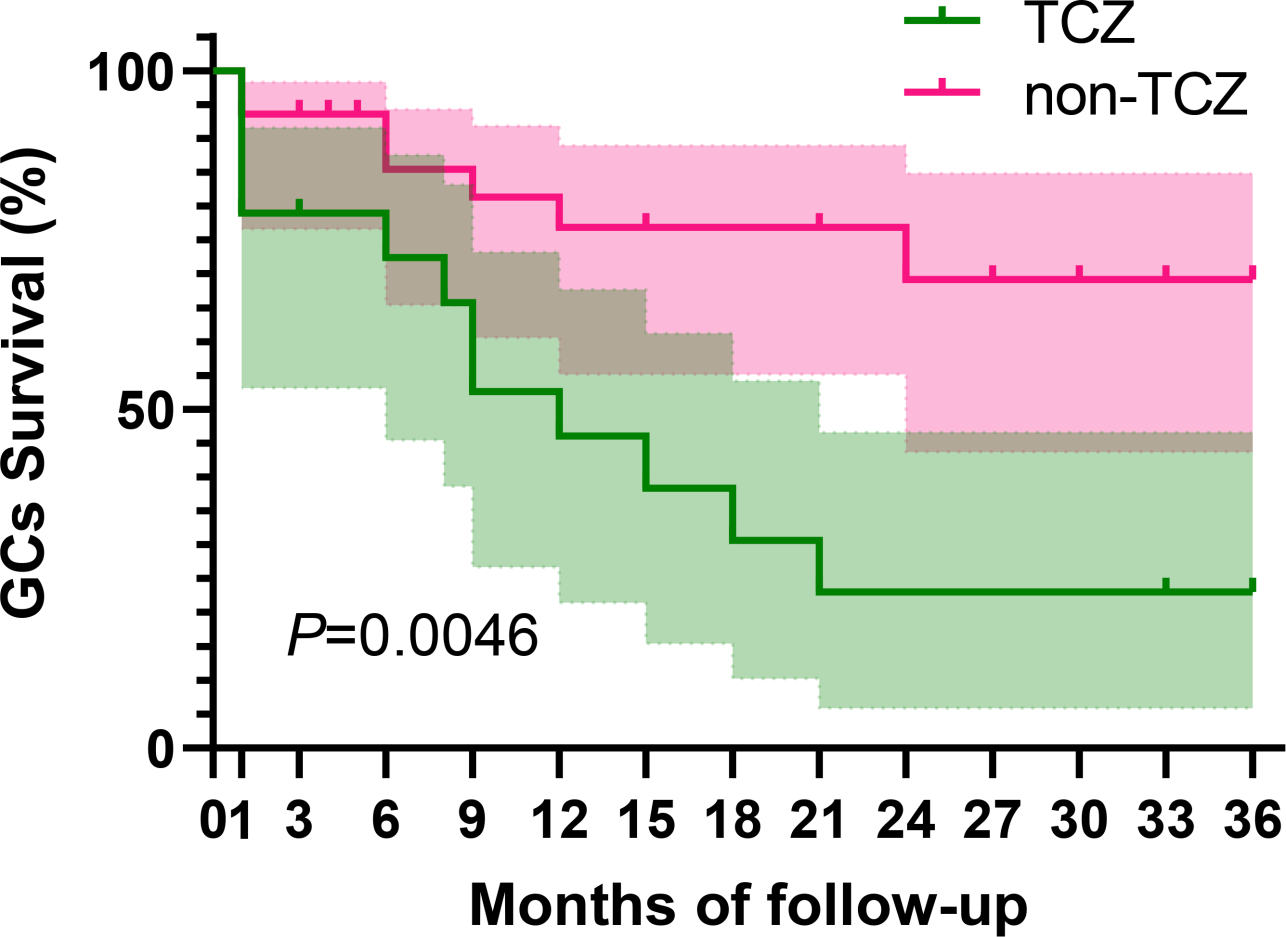
Glucocorticoid survival curves were illustrated using Kaplan-Meier analysis between the TCZ group and the non-TCZ group.

As shown in **Figure 3**, in the TCZ group, 5 patients (26.32%) in remission experienced recurrence, predominantly manifesting as non-arthralgia symptoms (80%). Conversely, in the non-TCZ group, 11 patients (40.74%) in remission reported recurrence, primarily characterized by arthralgia symptoms (63.64%). 4 patients in the non-TCZ group exhibited persistent arthralgia. Regarding glucocorticoids utilization, the dosage remained comparable between the TCZ and non-TCZ groups during the initial 12-month follow-up period (**Figure 2D**). However, at 12 months of follow-up, the proportion of patients using GCs in the non-TCZ group was consistently higher than that in the TCZ group (**Figure 4**). Moreover, by 36 months, the TCZ group demonstrated a significantly higher rate of GCs discontinuation in **Figure 4** (77.0% vs 30.9%, *P* = 0.0046).

### The utilization of csDMARDs and comparison of drug safety

**Figure 5:**
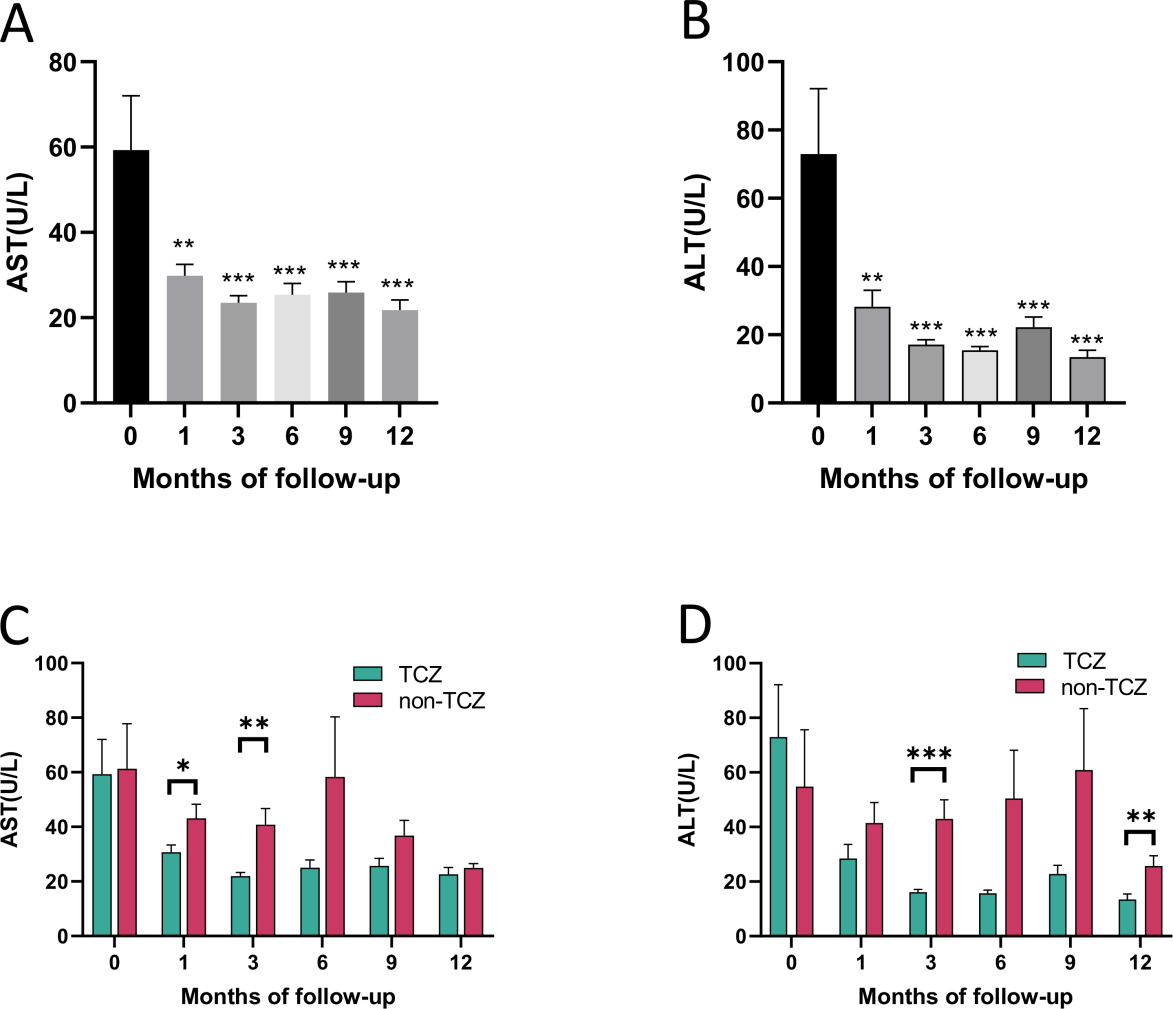
Comparison of transaminase levels between TCZ group and non-TCZ groups at different follow-up time points. **(A, B)** The levels of aspartate aminotransferase (AST) and alanine aminotransferase (ALT) were showed in the TCZ group at different follow-up time points. Compared to the initial treatment, *P < 0.05, **P < 0.01, ***P < 0.001. **(C, D)** The levels of AST and ALT were compared between TCZ group and non-TCZ group at different follow-up time points. *P < 0.05, **P < 0.01, ***P < 0.001.

Among the 50 AOSD patients, over 80% were administered methotrexate (MTX). The cumulative MTX dose over the 12-month period was comparable between the TCZ and non-TCZ groups (**Table 3**). The cumulative incidence of adverse events over the 12-month follow-up had significantly difference between two groups (**Table 4**). The overall rate of any adverse event in the TCZ group (21.1%) was markedly lower than that in the non-TCZ group (61.3%). Notably, one patient in the TCZ group received cyclosporine at initial diagnosis due to abnormal liver function, whereas six patients in the non-TCZ group required a switch to alternative immunosuppressants for the same reason (**Table 3**). During the first month, ALT and AST levels declined rapidly in the TCZ group (**Figures 5A, B**). By the third month, both AST (*P* < 0.01) and ALT (*P* < 0.001) were significantly lower in the TCZ group (**Figures 5C, D**). At the 12-month endpoint, transient liver enzyme elevations after initial normalization occurred in 10.5% (2/19) of TCZ patients (levels <50% of baseline) compared to 32.3% (10/31) of non-TCZ patients, with four of the latter showing elevations 2–5 times their baseline. Infections occurred in 10.5% (2/19) of the TCZ group and 16.1% (5/31) of the non-TCZ group (*P* = 0.893), including upper respiratory and urinary tract infections, which were resolved with appropriate treatment. Leukopenia was observed in one patient (3.2%) in the non-TCZ group only. Other infrequent events in the non-TCZ group included limb numbness, hypokalaemia, and tinnitus (one case, respectively). No new-onset diabetes or significant gastrointestinal events were recorded. One patient in the TCZ group developed MAS during the study period.

**Table 3:** Immunosuppressants and glucocorticoids in the TCZ group and the non-TCZ group.

Items	Treatment with tocilizumab group (n=19)	Treatment without tocilizumab group (n=31)	*P* value
MTX, n (%)	17(89.5)	26(83.9)	0.893
Cumulative dose of MTX, (mg)	464.12±105.06	482.31±88.96	0.886
LEF, n (%)	0	1(3.2)	–
CsA, n (%)	1(5.3)	0	–
HCQ, n (%)	0	3(9.7)	–
Thalidomide, n (%)	0	1(3.2)	–
Initial doses of MP (mg)	129.16±102.734	40.07±45.29	<0.001

MTX, Methotrexate; LEF, Leflunomide; CsA, Cyclosporine A; HCQ, hydroxychloroquine; MP, Methylprednisolone.

**Table 4:** Cumulative incidence of adverse events over the 12-month follow-up period in TCZ group and non-TCZ group.

Items	TCZ group (n=19)	non-TCZ group (n=31)	*P* value
Any Adverse Event, n (%)	4 (21.1)	19 (61.3)	0.013^*^
-Hepatic dysfunction, n (%)	2 (10.5)	10 (32.3)	0.160
-Leukopenia, n (%)	0 (0)	1 (3.2)	0.620
-Infection, n (%)	2 (10.5)	5 (16.1)	0.893
-Other Events^†^	0 (0)	3 (9.7)	–

^*^P<0.05; ^†^Other events included limb numbness, hypokalemia and tinnitus (1 case, respectively).

## Discussion

AOSD is a rare systemic inflammatory disorder, for which glucocorticoids serve as the primary therapeutic modality (8, 25). However, approximately 40-45% of patients encounter dependence or adverse effects (e.g., osteoporosis, diabetes) from GCs use (25, 26). csDMARDs, such as methotrexate, are employed adjunctively to mitigate GCs requirements (1, 8). Interleukin-6 (IL-6) plays a pivotal role in pathogenesis of AOSD and serves as a therapeutic target. IL-6 upregulates the expression of acute-phase reactants, such as ferritin and CRP, and promotes B-cell differentiation. Those induce systemic inflammation and end-organ damage. Additionally, IL-6 levels are closely correlated with the clinical manifestations of AOSD (2, 8–11, 13, 14, 16). Tocilizumab, an IL-6 receptor antagonist, has demonstrated efficacy in controlling disease activity and reducing glucocorticoids doses (14, 26). Despite this, the use of short-course high-dose glucocorticoids in combination with tocilizumab for rapid control of AOSD has not yet been reported. This retrospective study evaluated 50 patients meeting inclusion criteria to assess the efficacy, safety, and long-term GCs dose impact of this regimen. In this study, remission was defined as clinical inactive disease (CID), incorporating normalization of ESR or CRP to ensure accurate assessment of inflammatory activity in patients treated with tocilizumab. Tolerance thresholds for liver enzymes were integrated into the definition, enhancing its alignment with real-world clinical practice and improving the validity and comparability of outcomes. tocilizumab was initiated a median of 1 day after high-dose glucocorticoid therapy, reflecting a rapid combination approach. Our data demonstrated that this strategy effectively controlled AOSD activity and enabled timely glucocorticoid tapering upon achieving disease control, supporting its utility in clinical management (16, 26). Notably, within one month post-treatment initiation, inflammatory markers were significantly reduced and sustained in the TCZ group compared to the non-TCZ cohort throughout follow-up. Patients in the TCZ group exhibited more pronounced and rapid clinical improvement, with a significant decrease in Pouchot scores after one month. These results align with randomized controlled trial data, where most patients achieved an ACR50 response-indicating a 50% improvement-within the same timeframe (27). Although patients in the TCZ group had more non-arthralgia symptoms at baseline, during the 12-month follow-up period, the proportion of non-arthralgia symptoms that did not improve in the TCZ group was lower than that in the non-TCZ group. This phenomenon was consistent with the efficacy of tocilizumab in treating systemic symptoms (13). Among the patients in the TCZ group who did not achieve remission, a few cases had arthralgia, demonstrating the potential advantage of tocilizumab for patients with arthritis. The TCZ group demonstrated superior outcomes concerning symptom resolution and inflammatory marker reduction. During the 12-month follow-up, both remission and GCs discontinuation rates were notably high. The GCs discontinuation rate after 12 months of follow-up was used to assess the regimen’s potential for sustained GC tapering. Consequently, the observed GC discontinuation rates beyond 12 months (up to 36 months) are considered exploratory, reflecting preliminary evidence to inform the design of future prospective studies evaluating long-term steroid-free disease control. The combined approach of short-term high dose GCs with tocilizumab appears promising, offering enhanced disease control and potentially facilitating earlier GCs discontinuation. However, it remains lacking direct experimental evidence to support these findings, and more prospective studies are still required to validate the results.

Correct interpretation of this retrospective study was crucial. Our study was not designed to analyze the impact of glucocorticoid dosage in isolation, but rather to compare two clinical management strategies. The combination of tocilizumab with high-dose glucocorticoid pulse therapy operated on the principle of synergy: the rapid and broad-spectrum anti-inflammatory effects established a therapeutic window, thereby enabling the targeted and sustained action of tocilizumab. Our findings supported the overall clinical applicability and excellent early efficacy of this combined regimen, rather than attributing benefits to any single component. Although patients in the TCZ group received short-term high-dose GCs, their GCs dosage at one month was comparable to that of the non-TCZ group. Longitudinal follow-up data further revealed a significantly higher discontinuation rate of GCs in the TCZ group. The synergistic use of TCZ with short-term high-dose GCs facilitated more rapid disease stabilization and control, concurrently enabling a faster reduction in GCs dosage. In this investigation, patients treated with TCZ in conjunction with high-dose GCs exhibited expedited remission, reduced recurrence rates, and swift GCs tapering, consistent with existing literature (8, 9, 28). This treatment paradigm underscores the potential for optimizing therapeutic outcomes through targeted immunomodulation while minimizing long-term GCs exposure. The observed efficacy supports the hypothesis that early aggressive intervention with TCZ may confer substantial clinical benefits, particularly in managing systemic inflammation and mitigating the adverse effects associated with prolonged GCs use.

The baseline characteristics of patients in this study aligned with those documented in the literature for AOSD (3, 29). However, a higher incidence of skin rash and sore throat was noted in the TCZ group, potentially influencing treatment decisions. The one-month outcomes were comparable between the TCZ and non-TCZ groups. TCZ combined with short-term high-dose GCs demonstrated positive efficacy in managing non-articular symptoms. At treatment initiation, both the absolute count and percentage of neutrophils exceeded normal levels, corroborating previous findings (30, 31). Post one month of follow-up, neutrophil percentages normalized, showing a significant inter-group difference (32). Throughout follow-up, neutrophil levels remained stable. However, an upward trend was observed at nine months in the non-TCZ cohort, possibly due to complement influence on neutrophils (33) and TCZ’s broader impact on various cell types (32). Immunophenotype of AOSD has been proposed by some studies (31), warranting further comprehensive research. Patients receiving TCZ experienced swift symptom relief, whereas no significant differences in symptom alleviation rates were observed between groups by the end of follow-up. Our retrospective analysis indicated that TCZ combined with short-term high-dose GCs was safe and well-tolerated in the treatment of AOSD. Despite previous reports suggested that MTX may elevate liver enzyme levels (34) and IL-6 inhibitors could increase serum aminotransferase concentrations (27, 35), our findings demonstrated that transaminase levels in the TCZ group were significantly lower than those in the non-TCZ group during the initial three months of therapy. While transaminase levels remained lower in the TCZ group after three months, this difference was not statistically significant. Notably, the incidence of abnormal liver function was reduced in the TCZ group compared to the non-TCZ group. Nonetheless, regular monitoring of liver function remained essential when administering tocilizumab in combination with high-dose glucocorticoids. The cumulative 12-month MTX dose were not significantly different between two treatment groups. This finding robustly excluded differential MTX exposure as a plausible explanation for the higher incidence of liver dysfunction observed in the non-TCZ group. Therefore, the more favourable hepatic safety profile in the TCZ group was more likely attributable to the regimen’s superior efficacy in rapidly suppressing systemic inflammation. The comparable incidence of infections between two groups suggested that the combined induction strategy does not confer an additional infectious risk. At the one-year follow-up, CRP, SF, and N% were lower in the TCZ group but did not differ significantly from the non-TCZ group. These results suggested that tocilizumab may confer comparable long-term disease activity control to csDMARDs in AOSD patients. The prolonged disease control capacity of TCZ may parallel its effectiveness in other conditions such as Takayasu arteritis, necessitating extended follow-up (36). Early intervention with tocilizumab and short-term high-dose GCs appeared beneficial for rapid disease control, GCs doses reduction, and minimizing adverse effects. The significantly lower overall burden of adverse events occurred in the TCZ-based regimen. This study provided a novel real-world long-term evidence for the combination of TCZ with short-term high-dose GCs in treating AOSD, obtaining sustained outcomes including disease remission, long-term control of disease activity, and GCs tapering. Furthermore, our findings offered an actionable clinical decision support for the management of AOSD.

Our study has several limitations. Firstly, as a single-centre retrospective study, the design inherently limits the generalizability of the findings. Secondly, by comparing combination regimens, this study could not isolate the independent contribution of tocilizumab versus high-dose GCs pulses on therapeutic efficacy, thereby constraining mechanistic interpretation. Mechanistic studies exploring the synergistic effects of TCZ and GCs on inflammatory pathways (e.g., IL-6, CRP, and ferritin) will deepen our understanding of the combination therapy. Furthermore, the study had missing data and no predefined sample size. The lack of a blank control group hampers the possibility of controlling for spontaneous variations in disease activity related to the natural course of the disease. Moreover, due to the retrospective nature of this study and its limited sample size—an inevitable constraint in rare disease research—our ability to conduct comprehensive multivariate analyses to identify independent predictors of key clinical outcomes, including remission, relapse, and specific adverse events, was restricted. Although exploratory univariate analyses were performed, the statistical power was insufficient to adjust for potential confounders or to establish robust associations. In light of these constraints, we prioritized methodological rigor in primary intergroup comparisons to ensure validity and interpretability of the main treatment effects. Larger-scale prospective cohort studies are needed to ultimately determine patient characteristics or biomarkers associated with optimal efficacy of the combined induction therapy strategies evaluated in this study. Additionally, considering this study is observational, it is difficult to rule out the possibility of adverse results caused by pharmacological effects. In the future, prospective, multi-center randomized controlled trials are warranted to validate the causal efficacy and safety of TCZ combined with short-term high-dose GCs.

## Conclusion

Our findings indicated that the combination of TCZ with short-term high doses of GCs attributed to rapidly reduce disease activity and GCs dosage in patients with AOSD. This approach may optimize treatment strategies, potentially improving clinical outcomes while minimizing long-term GCs exposure. However, the durability of disease control and long-term safety of this regimen remain uncertain. More prospective studies are warranted to confirm these observations and refine therapeutic protocols for AOSD management.

## Data Availability

The original contributions presented in the study are included in the article/supplementary material. Further inquiries can be directed to the corresponding author.
